# Resveratrol Attenuates Heat Stress-Induced Luteal Injury Through Modulation of Oxidative Stress and Cytokine–Chemokine Inflammatory Networks in Pregnant Mice

**DOI:** 10.3390/antiox15040489

**Published:** 2026-04-14

**Authors:** Muhammad Tariq, Abdul Quddus, Kossinga Koulet André Saint Victor, Kebede Habtegiorgis Beshah, Yexiao Yan, Dagan Mao

**Affiliations:** College of Animal Science and Technology, Nanjing Agricultural University, Nanjing 210095, China; tariq@stu.njau.edu.cn (M.T.); abdulquddus@luawms.edu.pk (A.Q.);

**Keywords:** heat stress, resveratrol, corpus luteum, oxidative stress, transcriptomics, molecular docking

## Abstract

Heat stress (HS) affects female reproductive efficiency by disrupting redox homeostasis and activating inflammatory responses in the corpus luteum (CL), a metabolically active tissue essential for pregnancy maintenance. This study reveals the protective effect of resveratrol against HS-induced luteal injury in pregnant mice through the regulation of oxidative stress and cytokine–chemokine-mediated inflammatory and immune responses. The pregnant mice were divided into three groups: control, HS, and resveratrol +HS. Heat stress was applied at 40 ± 0.5 °C for 7 days, with resveratrol (10 mg/kg) given orally 2 h before exposure to HS. The results showed that heat exposure reduced serum total superoxide dismutase activity and increased malondialdehyde level, causing significant disruption of luteal morphology with cellular disorder and vacuolization, which was partially overcome by resveratrol pretreatment. Transcriptomic profiling showed that HS induced a strong immunological and inflammatory response, involving cytokine–cytokine receptor interaction and chemokine signaling. Resveratrol significantly attenuated HS-induced transcriptional changes. The RT-qPCR results showed that HS increased chemokine ligands (*Ccl11*, *Cxcl13*, *Tslp*) and cytokine receptors *(Ccr3*, *Ccr4*, *Ccr5*), which were suppressed by resveratrol. The chemokine-based inflammatory module is one of the most important regulatory properties of the HS response, according to the network analysis. Stable binding of resveratrol with major chemokine receptors was supported by molecular docking and molecular dynamics simulations. Collectively, HS induces oxidative, structural, and inflammatory alterations in luteal tissue, while resveratrol attenuates these changes by being associated with improved antioxidant status and suppression of cytokine–chemokine-mediated responses.

## 1. Introduction

The increase in ambient temperatures related to global climate change is a significant threat to the reproduction of mammals [1]. HS impairs systemic homeostasis and causes oxidative and inflammatory damage in various tissues, including the reproductive system [2]. Corpus luteum (CL) is a highly sensitive and well-vascularized gland that is especially vulnerable to hyperthermic damage in females [3,4]. In addition to its classical endocrine function, CL is currently considered to be the metabolically active and immunologically dynamic tissue. Luteal cells are highly metabolically active in mitochondria, with a strong association with vascular and immune regulatory mechanisms of tissue remodeling, progesterone synthesis, and luteal lifespan [5,6]. The redox and immune signaling imbalance leads to disruption of luteal integrity because of inflammatory and oxidative damage caused by stress [7].

High temperatures increase the activity of mitochondrial electron transport, which results in the overproduction of ROS. This leads to lipid peroxidation, DNA damage, and apoptosis of luteal cells [8,9]. HS conditions result in the imbalance between the ROS production and antioxidant capacity, which leads to oxidative stress, a key contributor to luteal structural and molecular alterations [10]. Studies in cattle have shown that HS compromises luteal vascular integrity, reduces progesterone synthesis, and disrupts cellular organization [3]. At the molecular level, oxidative stress activates redox-sensitive transcription factors, such as NF-κB and MAPKs, triggering the overexpression of cytokines and chemokines that promote inflammation and immune cell recruitment [11]. Accumulating evidence shows that cytokines, chemokines, and their related receptors are not merely byproducts of inflammation but also the early mediators of tissue damage [12]. Chemokine signaling in the corpus luteum occurs through the intensive control of immune cell recruitment, vascular permeability, and extracellular matrix remodeling [13]. These pathways could thus promote the acceleration of luteal malfunction and regression with prolonged activation by HS. This inflammatory environment exacerbates oxidative damage, forming a vicious cycle that accelerates luteal regression [14].

Natural polyphenols have attracted considerable attention due to their possible use in preventing oxidative and inflammatory damage [15]. Prior to single-target pharmacological compounds, phytochemicals usually have pleiotropic effects by acting as simultaneous antioxidative stress, inflammatory signaling, and receptor-mediated activators [16]. Such a multi-target effect is especially applicable in complicated pathologies caused by stress, including HS-related reproductive dysfunction, when redox imbalance, cytokine signaling, and tissue remodeling are occurring simultaneously [17]. Phytochemicals that can incorporate these regulatory layers, therefore, could have better protective potential. Resveratrol (3,5,4 7-trihydroxy-trans-stilbene) is a promising bioactive molecule with a wide range of antioxidant and anti-inflammatory effects [18]. It removes free radicals by its hydroxyl groups and enhances endogenous antioxidant defenses via the SIRT1/NRF2/HO-1 pathway [19]. Resveratrol prevents the activation of NF-KB and the production of pro-inflammatory cytokines, such as TNF-α, IL-1b, and IL-6 levels [20].

Noticeably, an inflammatory response in the corpus luteum is not a result of independent cellular processes but a process of interaction among various cytokines, chemokines, and their receptors [21]. Network-based analyses have become effective in the identification of central regulatory nodes that lead to inflammatory amplification of stress conditions [22]. The role of HS in remodeling these interaction networks, and the dynamic regulation of these networks by therapeutic interventions, is, therefore, crucial in understanding systems-level mechanisms in luteal dysfunction [23]. These cytokine–chemokine interactions provide coordinated signaling networks and non-independent molecular interactions, in which perturbation of a few major regulatory nodes can enhance inflammatory destruction during stressful conditions [24]. Conventional single-gene methods tend to be less effective at capturing these complicated patterns of interaction, especially in highly dynamic tissues, like the corpus luteum [25,26]. Network-based and systems biology methods are thus a more formalized way of making the central inflammatory regulators and their receptor-mediated signaling pathways identifiable [27,28]. The combination of transcriptomic profiling, network, and structural analysis will provide a good approach to find major molecular targets during HS-induced luteal damage [29,30].

Although the antioxidant and anti-inflammatory properties of resveratrol are well documented, the molecular mechanisms linking transcriptomic reprogramming to modulation of cytokine–chemokine networks and receptor-level interactions in the corpus luteum during pregnancy under HS conditions remain poorly defined. In particular, the systems-level and structural effects of resveratrol on inflammatory signaling networks within the CL have not been fully elucidated.

Therefore, the present study aimed to investigate the protective effects of resveratrol against HS-induced luteal damage in pregnant mice by integrating transcriptomic profiling, inflammatory network analysis, and molecular interaction modeling, supported by biochemical and histological validation.

## 2. Materials and Methods

### 2.1. Animals and Treatment

Thirty ICR female mice (7 weeks, 25–30 g) and fifteen males (12 weeks) were obtained from the Experimental Animal Center of Nanjing Agricultural University (Nanjing, China). Mice were housed under controlled conditions (22–24 °C, 12 h light/12 h dark cycle) with food and water ad libitum. All procedures were approved by the Institutional Animal Care and Use Committee of Nanjing Agricultural University (approval No. 20240507085) and followed the Guide for the Care and Use of Laboratory Animals (NRC, 2011).

Females in proestrus were mated overnight with males, and the presence of a vaginal plug was designated as day 1 of pregnancy (D1). The experimental unit was a single pregnant mouse divided into control (C), heat stress (HS), and resveratrol + heat stress (R+HS) groups (n = 10 each). Mice in the HS and R+HS groups were exposed to 40.5 ± 0.5 °C for 2 h daily (11:00–13:00) over seven consecutive days [31], whereas control mice remained at 22–24 °C. Mice in the R+HS group received resveratrol (10 mg/kg body weight, Beyotime Biotechnology, Shanghai, China) by oral gavage 2 h prior to heat exposure, dissolved in 0.5% carboxymethylcellulose. The dose, vehicle, and timing were selected based on established rodent pregnancy models [32]. Rectal temperature was measured before. Rectal temperature increased following heat exposure, confirming the physiological impact of the heat stress protocol, and after each exposure, using a thermistor probe, body weight was recorded daily [33]. On day 7 of pregnancy, mice were euthanized under isoflurane anesthesia; uterine and ovarian tissues were collected for analysis. Tissues were divided into transcriptomic sequencing, RT-qPCR validation, and H&E staining to evaluate gene expression and tissue morphology under HS and resveratrol treatment.

### 2.2. Measurement of the Oxidative Indexes

Blood was collected from each mouse and centrifuged at 3000× *g* for 15 min at 4 °C to get serum. Serum total superoxide dismutase (T-SOD) activity and malondialdehyde (MDA) concentrations were measured using commercial assay kits (Nanjing Jiancheng Bioengineering Institute, Nanjing, China) according to the manufacturer’s instructions.

### 2.3. Histopathological Analysis

The ovarian tissues were fixed in 4% paraformaldehyde for 24 h at 4 °C, dehydrated with graded ethanol series (70%, 80%, 90%, 95%, and 100%), cleared in xylene, and embedded in paraffin wax using standard histological methods. Paraffin-embedded blocks were sectioned at 5 μm thickness using a rotary microtome, and the sections were mounted on glass slides. For staining, the slides were deparaffinized in xylene, rehydrated through a descending ethanol series (100–70%), stained with hematoxylin for 10 min and eosin Y for 5 min, differentiated in lithium carbonate solution, dehydrated through ascending ethanol concentrations, cleared in xylene, and finally mounted with DPX [34]. Stained sections were examined under a light microscope (Olympus BX53, Olympus optical Co., Ltd., Tokyo, Japan) fitted with a digital camera (Nikon H550L, Nikon Corporation, Tokyo, Japan). Hematoxylin and eosin (H&E)-stained ovarian sections were evaluated based on established morphological criteria for ovarian and corpus luteum structure according to the INHAND guidelines [35]. Semi-quantitative scoring was performed for vacuolization, disorganization, inflammatory cell infiltration, and cell density using a 0–3 scale (0 = normal, 3 = severe). Five sections per group were analyzed. Scoring was conducted in a blinded manner to minimize bias, and the overall histological score was calculated as the sum of individual parameters and expressed as mean ± SEM.

### 2.4. Transcriptomic Analysis (RNA-Seq)

Total RNA was extracted from ovarian tissues using TRIzol reagent (Invitrogen, Carlsbad, CA, USA) according to the manufacturer’s instructions. The purity and concentration of RNA were determined using a NanoDrop spectrophotometer (Thermo Fisher Scientific, Waltham, MA, USA), and RNA integrity was assessed with an Agilent 2100 Bioanalyzer (Agilent Technologies, Santa Clara, CA, USA; RIN ≥ 7.0). Sequencing libraries were constructed from high-quality RNA samples using the Illumina TruSeq RNA Sample Preparation Kit (Illumina, San Diego, CA, USA). The Illumina NovaSeq 6000 sequencer (Illumina, San Diego, CA, USA) was used to perform paired-end sequencing (150 bp). Quality filtering of raw reads was constructed using FastQC (v0.11.9) and Trimmomatic (v0.39) to remove adapters, low-quality bases, and ambiguous reads. Clean reads were aligned to the mouse reference genome (GRCm39) using HISAT2 (v2.2.1), and gene-level counts were obtained with featureCounts (v2.0.1) [36]. Differential gene expression analysis between groups (C vs. HS and HS vs. R+HS) was performed using the DESeq2 package (v1.38.0) with thresholds of adjusted P (FDR) < 0.05 and |log2FoldChange| ≥ 1. Expression patterns were visualized using volcano plots and heatmaps generated with R software (v4.3.1). The Kyoto Encyclopedia of Genes and Genomes (KEGG) and Gene Ontology (GO) databases were used to perform the functional enrichment analysis of differentially expressed genes (DEGs) through the ClusterProfiler package (v4.6.2).

### 2.5. Real-Time Quantitative PCR Analysis

RT-qPCR was performed as previously described [37]. Total RNA was isolated from mouse ovarian tissues using RNA isolation reagent (R401-01, Vazyme Biotech Co., Ltd., Nanjing, China) and reverse transcribed to cDNA using the HiScript III RT SuperMix for qPCR (R323-01, Vazyme Biotech Co., Ltd., Nanjing, China) according to the manufacturer’s protocol. Each 20 μL of the PCR reaction was prepared as follows: 1 μL cDNA, 10 μL SYBR master mix, 8.2 μL nuclease-free water, and 0.4 μL each of forward and reverse primer pairs (10 μM). PCR was conducted on an ABI 7300 Fast Real-time PCR System (Applied Biosystems, Foster City, CA, USA) using ChamQ Universal SYBR qPCR Master Mix (Q711, Vazyme Biotech Co., Ltd., Nanjing, China). The qPCR cycling conditions included an initial hold at 95 °C for 5 min, followed by 40 cycles of 95 °C for 10 s and 60 °C for 30 s; then 95 °C for 15 s, 60 °C for 1 min, and 95 °C for 15 s. The RT-qPCR results were normalized to the reference gene glyceraldehyde 3-phosphate dehydrogenase (*Gapdh*). Relative expression levels of target genes were calculated based on the threshold cycle (Ct) values using the comparative 2^−ΔΔCt^ method. Each sample represented one independent biological replicate derived from mouse ovarian tissue, with five biological replicates per experimental group (n = 5). Each sample was analyzed in technical triplicate. The sequences of the target gene are shown in Table 1.

### 2.6. Network Analysis

Protein–protein interaction (PPI) network analysis was conducted to investigate the interaction map of cytokine genes and chemokine genes that were significantly dysregulated under HS. The organism was set to Mus musculus, and the DEGs involved in the cytokine–cytokine receptor interaction pathway were imported into the STRING database (version 11.5; https://string-db.org, accessed on 26 December 2025). Interactions with a confidence score greater than 0.9 were considered to ensure the reliability of the network. Isolated nodes were eliminated to enhance network clarity. The interaction network was exported as a TSV file and visualized using Cytoscape software (version 3.9.1). Major regulatory nodes were identified through topological analysis of the network. The cytoHubba plugin was used to identify hub genes using degree centrality and the maximal clique centrality (MCC) ranking method. Genes with the highest centrality scores were considered central regulators of the HS-induced inflammatory network.

### 2.7. Molecular Docking Analysis

Molecular docking studies were performed to clarify structural insights into chemokine–receptor interactions and to assess the possible binding of resveratrol to key inflammatory receptors identified through both transcriptomic and network analyses. Docking targets were selected from hub genes identified in the PPI network based on degree centrality and maximal clique centrality (MCC) and prioritized according to high-confidence STRING interaction scores, established chemokine ligand–receptor pairing, and biological relevance and further refined based on favorable docking performance, including strong binding affinity and stable interaction profiles. Protein–protein docking was conducted to explore the interactions between central chemokines and their receptors, including CCL11 (UniProt ID: P48298)–CCR3 (UniProt ID: P51678), CXCL12 (UniProt ID: P40224)–CXCR4 (UniProt ID: P70658), and CXCL10 (UniProt ID: P17515)–CXCR3 (UniProt ID: O88410). Three-dimensional structures of chemokines and receptors were obtained from UniProt and the Protein Data Bank (PDB). Protein structures were obtained by removing water molecules, adding hydrogen atoms, and optimizing protein geometry. HDOCK web server (version 1.1; http://hdock.phys.hust.edu.cn, accessed on 4 January 2026) was used to perform the docking; this system integrates template-based modeling with ab initio docking methods. Docking models with the lowest binding energies were ranked at the top, analyzed, and visualized in terms of interaction and structure.

Small-molecule docking was conducted to investigate the molecular mechanism underlying resveratrol-induced inflammatory signaling through its interaction with major chemokine receptors (CCR3 (UniProt ID: P51678), CXCR3 (UniProt ID: O88410), and CXCR4 (UniProt ID: P70658)). The chemical structure of resveratrol in 2D was created using ChemDraw Professional (version 20.0, PerkinElmer, Waltham, MA, USA) and then transformed into a 3D, energy-minimized structure. Protein preparation involved optimizing protonation states and eliminating unnecessary molecules. Docking was performed using AutoDock Vina (version 1.1.2), and binding affinities were estimated based on docking scores. The most preferable binding poses were selected for further interaction analysis and subjected to molecular dynamics simulations to assess the binding stability under dynamic conditions.

### 2.8. Molecular Dynamics Simulation

The stability of the docked resveratrol–protein complexes was assessed using the Desmond molecular dynamics simulation package (Schrödinger, LLC., New York, NY, USA; version 2021-4). Molecular dynamics (MD) simulations were performed on the docked complex at a constant temperature of 303.3 K using an NPT ensemble (constant number of particles, pressure, and temperature). The default Desmond barostat was used to maintain the pressure at 1 bar. All systems were simulated to 100 ns, and the trajectories’ coordinates were recorded every 1000 simulation steps for subsequent analysis. The dynamic behavior and structural stability of the protein–ligand complexes were estimated by calculating the root mean square deviation (RMSD) of the protein backbone, the root mean square fluctuation (RMSF) of each individual residue, and the average RMSD per frame from the generated trajectory files. These parameters were compared to measure the conformational stability and flexibility of the complexes during the simulation period.

### 2.9. Statistical Analysis

All data are presented as mean ± SEM. GraphPad Prism software (version 9.5.1; GraphPad Software, San Diego, CA, USA) was used to carry out the statistical analysis. Differences among experimental groups were analyzed using one-way analysis of variance (ANOVA) followed by Tukey’s post hoc multiple comparison test. Data was tested for normality and homogeneity of variance prior to analysis. *p*-value < 0.05 was considered statistically significant.

## 3. Results

### 3.1. Effect of HS and Resveratrol on Serum Antioxidant Status and Lipid Peroxidation in Mice

**Figure 1:**
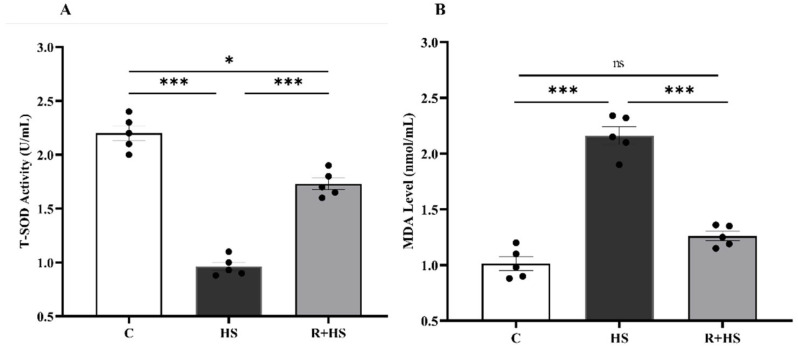
Effects of heat stress and resveratrol on serum antioxidant status and lipid peroxidation in pregnant mice. (**A**) T-SOD activity. (**B**) MDA level. Data are presented as mean ± SEM, with individual data points representing each animal (n = 5 per group). Statistical significance was determined by one-way ANOVA followed by Tukey’s post hoc test (* *p* < 0.05, *** *p* < 0.001; ns, not significant). C: control group; HS: heat stress group; R+HS: resveratrol + heat stress group.

Serum T-SOD activity and MDA level were measured. HS significantly decreased serum T-SOD activity (Figure 1A) and increased MDA level (Figure 1B) compared to the control group. In contrast, resveratrol pretreatment significantly increased T-SOD activity and reduced MDA level in comparison to the HS group.

### 3.2. Histopathological Evaluation of Heat Stress and Resveratrol-Treated Corpus Luteum

**Figure 2:**
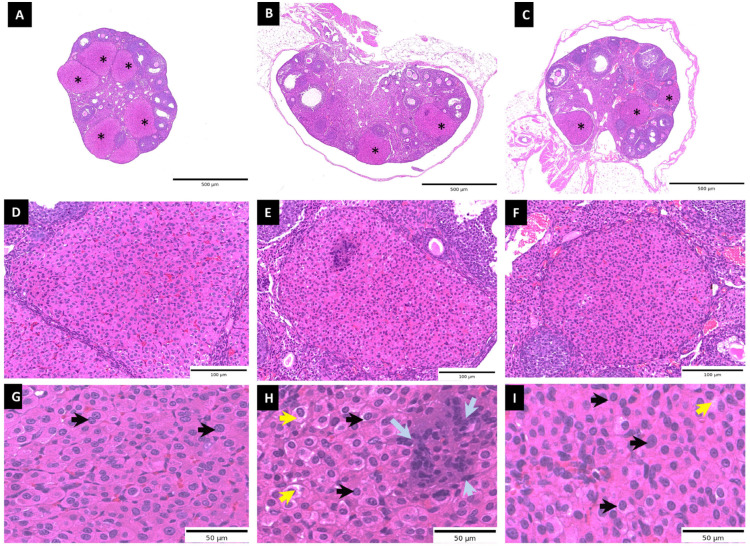
Histological evaluation of ovarian tissue under heat stress and resveratrol treatment. Representative H&E-stained sections of control (**A**,**D**,**G**), heat stress (HS) (**B**,**E**,**H**), and HS + resveratrol (**C**,**F**,**I**) groups at low (**A**–**C**), medium (**D**–**F**), and high magnification (**G**–**I**). Asterisks (*) indicate CL. The control group shows normal luteal architecture with densely packed luteal cells (black arrows) and a well-developed vascular network, indicative of normal physiology. The HS group exhibits histological alterations, including cytoplasmic vacuolization (yellow arrows), cellular disorganization, inflammatory cell infiltration (gray arrows), and reduced vascular integrity. These changes are attenuated in the HS + resveratrol group, which shows improved cellular organization, reduced vacuolization, and partial restoration of vascular structure.

A greater number of well-defined corpora lutea (CL) and preserved ovarian architecture were observed in the control group (Figure 2A,D,G). Luteal cells were densely packed with a compact arrangement, homogeneous cytoplasm, and distinct nuclei (black arrows), consistent with normal luteal morphology. In contrast, the heat stress (HS) group showed a reduced number of corpora lutea and disrupted luteal architecture (Figure 2B,E,H). Histological alterations included cellular disorganization, enlarged intercellular spaces (yellow arrows), cytoplasmic vacuolization, nuclear condensation, and inflammatory cell infiltration (gray arrows), indicating tissue injury associated with heat stress. In the HS + resveratrol group, luteal structural integrity was partially improved (Figure 2C,F,I). Luteal cells exhibited improved organization and more preserved nuclear morphology compared to the HS group, although slightly increased intercellular spaces were still observed. Semi-quantitative histological scoring further supported these observations (Table 2), demonstrating a significant increase in tissue alteration in the HS group compared to control (*p* < 0.05), while resveratrol treatment significantly reduced histological damage compared to the HS group (*p* < 0.05), although values remained elevated relative to control (*p* < 0.05).

### 3.3. Transcriptomic Profiling Reveals Heat Stress-Induced Activation of Cytokine–Chemokine Interaction in the Ovary

**Figure 3:**
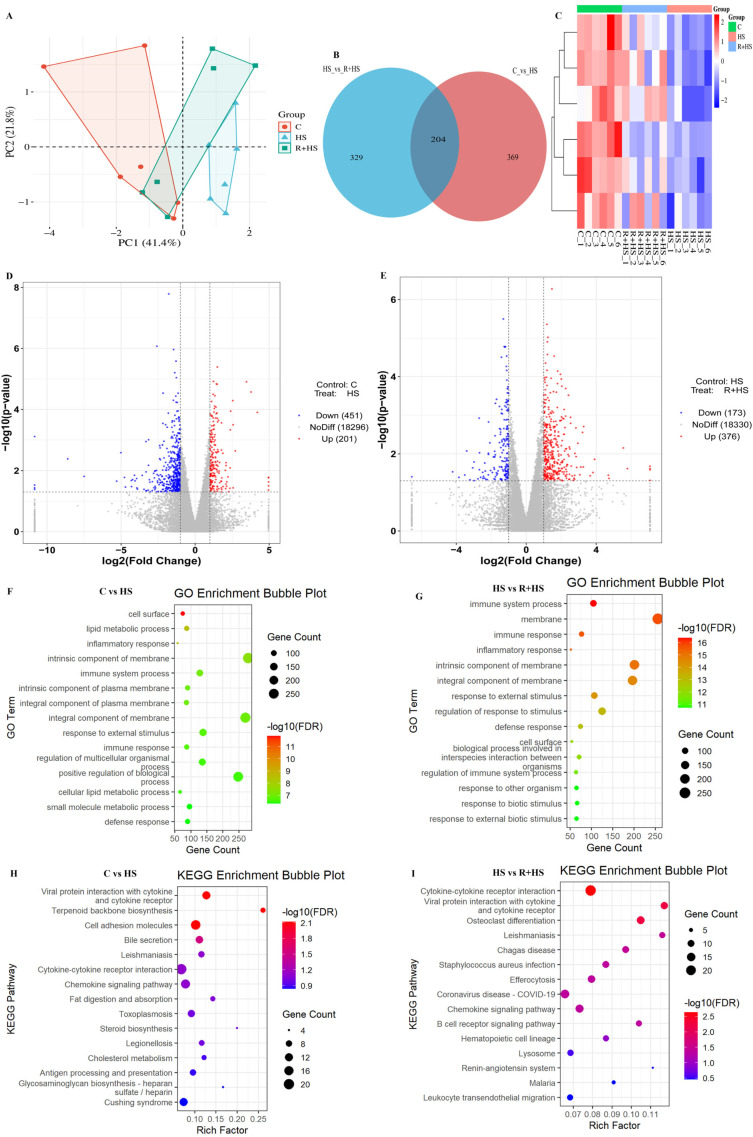
Heat stress and resveratrol regulation of the expression of genes in mouse ovarian tissue by transcriptomic profiling. (**A**) Principal component analysis (PCA) of the clusters of samples of the control (**C**), heat stress (HS), and resveratrol and heat stress (R+HS) groups. (**B**) Venn diagram of shared and unique differentially expressed genes (DEGs) between C vs. HS and HS vs. R+HS comparisons. (**C**) Heatmap depicting a hierarchical clustering of the cytokine/chemokine-related DEGs, with rows corresponding to the genes and columns corresponding to each sample within the treatment groups (C, HS, and R+HS). Relative gene expressions are measured by color intensity. Volcano plots of C vs. HS (**D**) and HS vs. R+HS (**E**) of DEGs. (**F**,**G**) Gene Ontology (GO) enrichment analysis of DEGs between (C vs. HS) (**F**) and (HS vs. R+HS) (**G**). (**H**,**I**) Kyoto Encyclopedia of Genes and Genomes (KEGG) pathway enrichment analysis of DEGs between C vs. HS (**H**) and HS vs. R+HS (**I**). RNA-seq analysis was performed using n = 6 biological replicates per group.

RNA-seq was used to assess the transcriptional alterations of heat stress and the modulation capabilities of resveratrol on the ovarian tissue. PCA indicated that there was clear separation among groups (Figure 3A), with PC1 and PC2 accounting 41.4% and 21.8% of the total variance, respectively. The Venn analysis showed that there were 204 overlaps of DEGs in the C vs. HS comparison and HS vs. R+HS (Figure 3B). Moreover, 369 DEGs were unique to the C vs. HS comparison, and 329 were unique to the HS vs. R +HS comparison, which suggests a heat stress-specific transcriptional response and a resveratrol-modulated transcriptional response. Among the overlapping DEGs, some of the key inflammatory and chemokine-related genes, including *Ccl11*, *Cxcl12*, *Cxcl13*, and *Tslp,* were highly upregulated in the HS group relative to the control group, as well as the receptors, *Ccr3*, *Ccr4*, and *Ccr5*. These genes were consistently attenuated in the R+HS group, indicating suppression of heat stress-induced inflammatory signaling. Hierarchical clustering of cytokine- and chemokine-related DEGs revealed distinct expression patterns among the experimental groups. The HS group samples were clearly separated from the control group, while the samples of the R+HS group were closer to the control group, which showed partial normalization of transcriptional changes after resveratrol treatment (Figure 3C). The volcano plot revealed 451 downregulated and 201 upregulated genes in HS compared with the C group (Figure 3D). The comparison of HS vs. R+HS, however, revealed 376 upregulated and 173 downregulated genes in the R+HS group (Figure 3E). Further, the comparison between HS and R+HS analysis revealed that a group of genes was characterized by relatively higher expression in the resveratrol-treated group than in HS, indicating some normalization of transcriptional responses towards control levels. GO enrichment revealed that DEGs caused by HS were mainly engaged in inflammatory and immune events, especially cytokine signaling pathways, and enriched membrane/cell surface elements (Figure 3F). These immune-related GO enrichments were markedly attenuated in the R+HS group compared with the HS group (Figure 3G). KEGG analysis further supported the finding that compared to the HS group, there was strong enrichment of cytokine–cytokine receptor interaction and chemokine signaling in the C vs. HS group (Figure 3H), which was alleviated in the HS vs. R+HS group (Figure 3I).

### 3.4. RT-qPCR Validation of Heat Stress and Resveratrol-Responsive Genes

**Figure 4:**
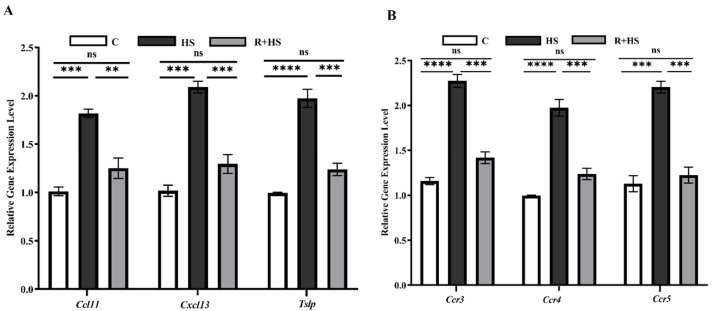
RNA-seq validation by RT-qPCR analysis of the selected differentially expressed genes in mouse ovarian tissue. (**A**) Relative mRNA expression levels of cytokine/chemokine ligands (*Ccl11*, *Cxcl13*, *Tslp*). (**B**) Relative mRNA expression levels of chemokine receptors (*Ccr3*, *Ccr4*, *Ccr5*) in control (C), heat stress (HS), and resveratrol + heat stress (R+HS) groups. Data are presented as mean ± SEM (n = 5 biological replicates per group, with technical triplicates). Statistical analysis was performed using one-way ANOVA followed by Tukey’s post hoc test. Data were tested for normality and homogeneity of variance prior to analysis. Overall ANOVA *p*-values are indicated for each panel. Significance levels are defined as ** *p* < 0.01, *** *p* < 0.001, **** *p* < 0.0001 and ns, not significant.

RT-qPCR was performed to validate the expression of selected genes in the ovary. Consistent with the RNA-seq results, HS significantly increased the mRNA expression levels of inflammatory cytokine ligands, such as *Ccl11*, *Cxcl13*, and *Tslp,* in comparison with the control group (Figure 4A). Treatment with resveratrol significantly reduced the mRNA expression of cytokines in R+HS groups compared with the HS group. Similarly, the expression level of chemokine receptors *Ccr3*, *Ccr4,* and *Ccr5* increased in the HS mice compared to controls (Figure 4B), which were significantly reduced by resveratrol supplementation. The control and resveratrol-treated groups show no significant differences in all the genes studied.

### 3.5. Heat Stress Activates Redox-Driven Chemokine Network and Resveratrol Suppresses Inflammatory Hub Network Analysis

**Figure 5:**
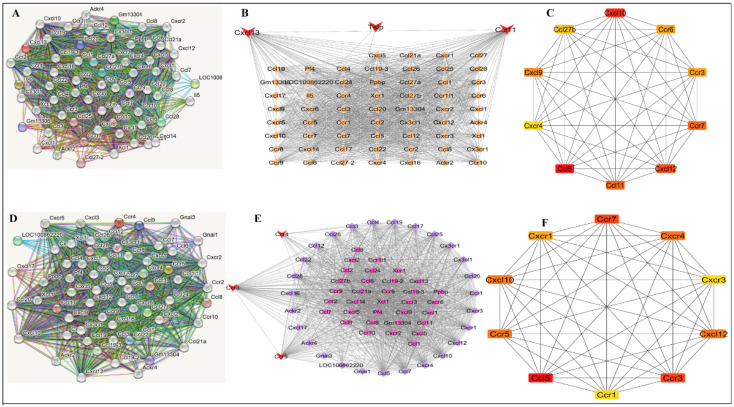
PPI network analysis of HS and resveratrol-responsive genes. (**A**–**C**) Chemokine-centered hubs for upregulated genes in the HS group compared with the C group. (**D**–**F**) Chemokine-centered hubs for downregulated genes in the R+HS group compared with the HS group.

A highly interconnected network of PPI was induced by HS (Figure 5A), which is indicative of coordinated activation of inflammatory signaling. This network was centered on chemokine ligands and chemokine ligand receptors (Figure 5B). Hub analysis revealed that Ccl11, Cxcl10, Cxcl12, Ccr3, Ccr4, and Ccr5 were the central nodes in terms of degree and maximal clique centrality (Figure 5C). Conversely, the resveratrol-modulated network (genes downregulated in R+HS compared to HS) was characterized by smaller network size and connectivity (Figure 5D,E) and less centrality of inflammatory hub genes (Figure 5F), implying inhibition of chemokine-based inflammatory signaling.

### 3.6. Molecular Docking

#### 3.6.1. Protein–Protein Docking Analysis of Key Chemokine–Receptor Complexes

Protein–protein docking was performed for key chemokine–receptor pairs shown from transcriptomic and network analyses (CCL11–CCR3, CXCL12–CXCR4, and CXCL10–CXCR3) to explore the structural basis of chemokine-driven signaling under heat stress. All complexes showed favorable docking scores, large interface areas, and stable interaction energies (Table 3). Among them, CCL11–CCR3 exhibited the strongest docking affinity and the largest interface area, supporting its central role in luteal inflammation.

#### 3.6.2. Structural Visualization of Chemokine–Receptor Docking Complexes

**Figure 6:**
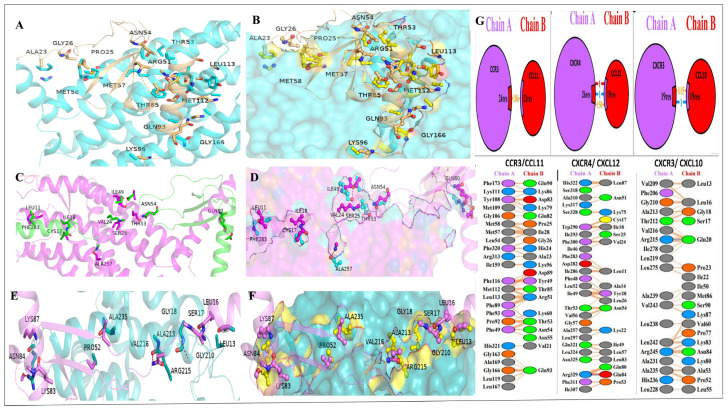
Chemokine–receptor complexes visualization of protein–protein docking. (**A**–**F**) Ribbon and surface views of the CCL11-CCR3 (**A**,**B**), CXCL12-CXCR4 (**C**,**D**), and CXCL10-CXCR3 (**E**,**F**) Receptors: cyan (CCR3/CXCR3) and magenta (CXCR4); chemokines: tan (CCL11), green (CXCL12), magenta (CXCL10), and yellow (surface views). Interacting residues shown as sticks with labels (**G**) Residue-level interaction networks showing interface residue counts (24/21, 26/19, 19/19) and interaction numbers (186, 150, 115). Top: schematics with purple ovals (Chain A/receptors) and red ovals (Chain B/chemokines). Bottom: contact tables with color-coded residue properties—purple (aromatic), blue (basic), red (acidic), green (polar), orange (hydrophobic), gray (other), yellow (cysteine).

The docking conformations with the highest rank were visualized to evaluate the binding orientation and interface properties of the chemokine–receptor complexes of interest. Ribbon and surface representations revealed that CCL11 had a stable docking position at the binding interface of CCR3 and was in close contact with interface residues (Figure 6A,B). Similarly, CXCL12 exhibited a stable binding orientation with CXCR4 (Figure 6C,D), while CXCL10 exhibited consistent docking at the interaction site of CXCR3 (Figure 6E,F), which was supported by high surface complementarity and a clear binding interface. The interaction mapping revealed that the complex stabilization was predominantly mediated by non-covalent interactions, such as hydrogen bonds, salt bridges, disulphide bonds, and non-bonded contacts, which included residues of both the receptor (Chain A) and the chemokine ligand (Chain B) (Figure 6G).

### 3.7. Molecular Docking Analysis of Resveratrol with Key Chemokine Receptors and Inflammatory Hub Proteins

#### 3.7.1. Molecular Docking Analysis of Resveratrol with Key Chemokine Receptors

Molecular docking was done to determine whether resveratrol might directly bind major inflammatory chemokine receptors (*CCR3*, *CXCR3,* and *CXCR4*) that were downregulated after resveratrol treatment. Resveratrol had similar binding affinities to the three receptors (−7.2 to −6.8 kcal/mol, Table 4) and assumed stable conformations in their binding pockets. Interaction profiling revealed that hydrogen bonding and hydrophobic contacts favored binding, which implies that resveratrol may act as a multi-target modulator of chemokine receptor-based signaling as a multi-target.

#### 3.7.2. Structural Features of Resveratrol Binding to Chemokine Receptors

**Figure 7:**
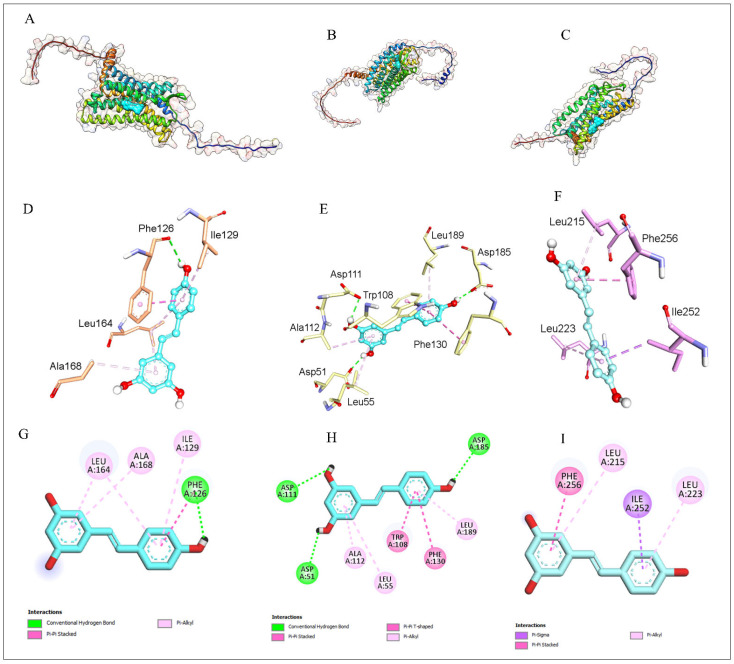
Structural visualization of resveratrol binding to chemokine receptors. Resveratrol interacts with CCR3, CXCR3, and CXCR4. (**A**–**C**) Ribbon/surface views. (**D**–**F**) Binding site residues. (**G**–**I**) 2D interaction maps.

Structural visualization proved that resveratrol has a binding site in the transmembrane pockets of CCR3, CXCR3, and CXCR4 (Figure 7A–C). Each binding site had stable ligand-specific orientations. The binding was stabilized by a complex of hydrogen bonds and hydrophobic interactions between a key aromatic and aliphatic residue, as shown by residue-level analysis (Figure 7D–F). Regularly, 2D interaction maps showed receptor-specific contact motifs, such as π–pi stacking and pi–alkyl contacts, showing stable binding of resveratrol to all receptors (Figure 7G–I).

### 3.8. Molecular Dynamics Simulation Analysis

#### 3.8.1. Molecular Dynamic Simulation Analysis of the Resveratrol–Chemokine Receptor

**Figure 8:**
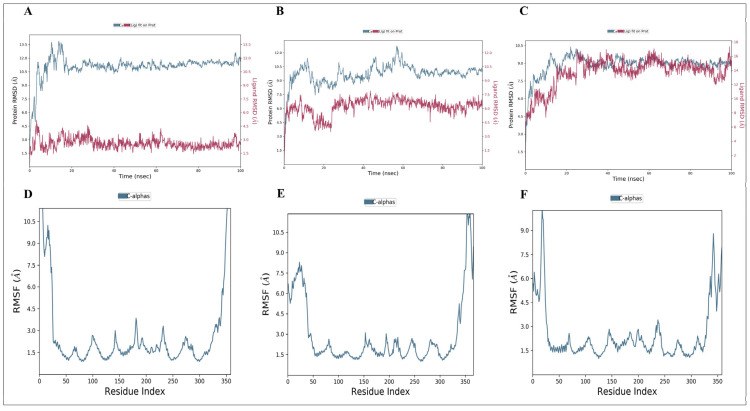
Molecular dynamics simulation analysis of resveratrol–chemokine receptor complexes. (**A**–**C**) RMSD of protein backbone and bound resveratrol of the resveratrol–CCR3, resveratrol–CXCR3, and resveratrol–CXCR4 complexes after a 100 ns simulation. (**D**–**F**) RMSF analysis of residue-level flexibility of CCR3, CXCR3, and CXCR4 in complex with resveratrol.

MD simulations of resveratrol–CCR3, resveratrol–CXCR3, and resveratrol–CXCR4 were conducted at 100 ns to assess complex stability. RMSD profiles showed that all systems achieved the equilibrium in the initial stages of the simulation and stayed constant during the simulation (Figure 8A–C). Resveratrol–CCR3 had the lowest RMSD, which implies the greatest structural stability, and resveratrol–CXCR4 had a short period of initial adjustment followed by stabilization. RMSF analysis showed that flexibilities were predominantly confined to terminal and loop domains, while residues in the ligand binding pockets were relatively stable (Figure 8D–F) to maintain the ligand binding.

#### 3.8.2. Binding Free Energy Decomposition and Key Residue Contributions

**Figure 9:**
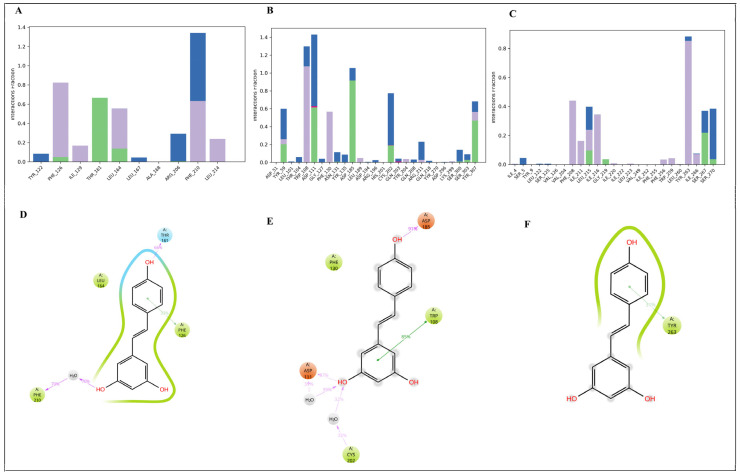
Dissociation of per-residue binding free energy of resveratrol–chemokine receptor complexes. (**A**–**C**) Profile of energy contribution with resveratrol–CCR3 (**A**), CXCR3 (**B**), and CXCR4 (**C**) as functions of their residue. Blue bars show the contribution of van der Waals energy, green bars indicate the contribution of electrostatic energy, purple bars show the contribution of polar solvation energy, red bars show the contribution of non-polar solvation energy, and the black line shows the contribution of total binding free energy per residue unit. (**D**) Two-dimensional interaction map between resveratrol and CCR3. (**E**) Two-dimensional interaction map of resveratrol with CXCR3, showing binding within the ligand-binding pocket through hydrogen bonding and hydrophobic interactions with key amino acid residues. (**F**) Two-dimensional interaction map between resveratrol and CXCR4.

The decomposition in per-residue binding free energy was used to describe resveratrol binding to chemokine receptors. CCR3/resveratrol binding was predominantly governed by van der Waals and non-polar energies, with Phe126, Ile129, and Leu164 making the most significant contributions (Figure 9A). In CXCR3, the residue contributions were more distributed, van der Waals and electrostatic, which showed the involvement of polar and non-polar residues (Figure 9B). In the case of CXCR4, van der Waals and non-polar solvation effects were predominant at the binding sites between the residues that form a hydrophobic pocket (Figure 9C). Interaction maps using a two-dimensional format further depicted residue-level contacts on CCR3, CXCR3, and CXCR4 (Figure 9D–F).

#### 3.8.3. Protein–Protein Molecular Dynamics Simulation

**Figure 10:**
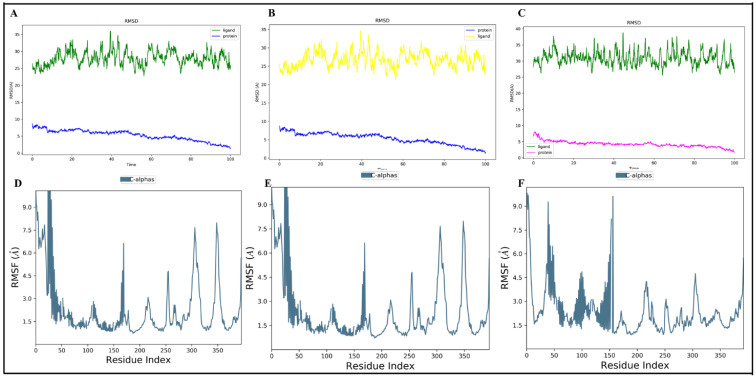
Chemokine–receptor complexes simulation by molecular dynamics. (**A**–**C**) RMSD profiles of CCL11-CCR3 (**A**), CXCL12-CXCR4 (**B**), and CXCL10-CXCR3 (**C**) over 100 ns. (**D**–**F**) RMSFD profiles of C α atoms of CCR3 (**D**), CXCR4 (**E**), and CXCR3 (**F**).

MD simulations of *CCL11-CCR3*, CXCL12-CXCR4, and CXCL10-CXCR3 complexes were carried out. The profiles of RMSD of CCL11-CCR3 (Figure 10A), CXCL12-CXCR4 (Figure 10B), and CXCL10-CXCR3 (Figure 10C) were maintained at 100 ns. RMSF revealed minimal changes in binding regions to ligands and increased flexibility in terminal regions and extracellular loops in CCL11-CCR3 (Figure 10D), CXCL12-CXCR4 (Figure 10E), and CXCL10-CXCR3 (Figure 10F). These findings verify the existence of stable chemokine–receptor interactions in dynamic conditions.

## 4. Discussion

The current study demonstrates that HS triggers oxidative stress, transcriptional reprogramming, and structural disorganization in the ovary of pregnant mice and that resveratrol supplementation attenuates these pathological alterations. By integrating biochemical evaluation, transcriptomics profiling, network analysis, molecular docking, and molecular dynamics simulation, this study provides integrated insights into potential mechanisms of how resveratrol alleviates HS-induced luteal impairment.

One of the central findings of this study is that HS triggers a strong oxidative imbalance, as evidenced by decreased antioxidant activity and increased lipid peroxidation. Similar decreases in antioxidant enzyme activity and increases in oxidative damage markers have been reported in heat-stressed ovarian and luteal tissues across various mammalian species, with excess reactive oxygen species (ROS) disrupting subcellular integrity and steroidogenic capacity [38]. Heat stress in mammals is associated with increased core body temperature, oxidative stress, systemic inflammation, and impaired organ function, which collectively contribute to reproductive dysfunction [39]. The ultrastructural alterations observed in the CL, including the subcellular swelling and architecture disruption, are consistent with the previous experimental results that HS has been reported to impair luteal activity and progesterone production in previous studies [40,41]. A combination of these results supports the idea that oxidative stress is a key upstream driver of heat-induced luteal injury.

Heat-induced oxidative stress was attenuated by resveratrol pretreatment, with significant improvement of antioxidant capacity and reduced lipid peroxidation. In ovarian granulosa and luteal cells, experimental studies have shown that resveratrol enhances mitochondrial antioxidant defenses, stabilizes mitochondrial membrane potential, and decreases intracellular ROS [42,43]. In vivo experiments further indicate that resveratrol supports the structural integrity of reproductive tissues under stress conditions [44,45]. Extending these observations, our findings suggest that resveratrol exerts protective effects on the corpus luteum during pregnancy primarily through the improvement of redox homeostasis, rather than nonspecific cytoprotective actions.

Heat stress caused significant histopathological changes in the corpus luteum, causing disturbed cellular order, cellular vacuolization, and cellular nuclear condensation, which denoted structural defects and compromised luteal integrity. These pathologies show the presence of oxidative and inflammatory damage in luteal tissue [46,47]. Resveratrol pretreatment preserved the luteal morphology, which enhanced cellular organization and nuclear integrity. This aligns with the findings that resveratrol improves the reproductive tissue resistance to oxidative stress and has been reported to promote steroidogenic activity [48,49]. Collectively, resveratrol attenuates HS-induced luteal injury by improving the redox balance and inhibiting inflammation.

In addition to oxidative damage and histopathological changes, transcriptomic profiling has shown that HS causes an inflammatory gene expression pattern in the corpus luteum (CL), with a strong enrichment of cytokine–cytokine receptor interaction pathways and inflammatory Gene Ontology categories. Importantly, RNA-seq revealed that chemokine ligands and receptors such as *Ccl11*/*Ccr3*, *Cxcl10*/*Cxcr3,* and *Cxcl12*/*Cxcr4* were upregulated significantly, which is an indication of chemokine-centered inflammatory networks activation. These transcriptomic data were also supported by RT-qPCR, which revealed significant changes in mRNA expression levels of *Ccl11*, *Cxcl13*, and *Tslp* and the corresponding receptors *Ccr3*, *Ccr4*, and *Ccr5,* upregulated by the HS group relative to controls, which further justified the reliability of the sequencing data and supported the existence of an amplified inflammatory response. As has been reported in previous studies, the upregulation of inflammatory cytokines and chemokines by HS in reproductive tissues leads to the recruitment of immune cells and the boosting of inflammatory cascades [50,51]. The overexpression of chemokine signaling in the CL has been associated with pathological luteal regression and tissue repair dysregulation [52,53,54]. Although a balance between normal luteal growth and immune tolerance in the body during pregnancy requires the function of controlled inflammatory signaling [55], our results reveal that HS disrupts this balance, and resveratrol alleviates such a pathological change by suppressing the coordinated cytokine–chemokine signaling.

PPI network analysis also showed that HS induces a chemokine-centered inflammatory network with chemokine ligands and receptors connected in highly interlinked hub-nodes. This finding is also compatible with previous experimental network analyses, suggesting that inflammatory pathology may arise from the coordinated activation of key network nodes rather than isolated gene dysregulation [56,57]. The prominence of chemokine hubs in the network shows that HS accelerates the amplification of inflammatory processes through interconnected signaling cascades. Conversely, resveratrol treatment decreased network connectivity as well as the hub centrality, demonstrating that it suppresses coordinated inflammatory signaling rather than merely downregulates a single gene. Experimental models of inflammatory and metabolic disorders have also reported similar network-level anti-inflammatory effects of resveratrol [58,59]. Consistent with the modification of network topology, KEGG pathway enrichment of network-derived genes further showed significant cytokine–cytokine receptor interaction and chemokine signaling pathways in response to HS and attenuated by resveratrol.

Molecular docking and molecular dynamics simulations were performed to further investigate the possible molecular mechanisms underlying resveratrol-mediated inhibition of inflammatory signaling. Protein–protein docking confirmed the stable interactions between major chemokine ligands and their receptors, consistent with established chemokine biology [60,61]. Resveratrol docking showed desirable binding conformations with CCR3, CXCR3, and CXCR4, primarily mediated by non-covalent interactions, including hydrogen bonding and hydrophobic contacts. The stability of such complexes over time was supported through molecular dynamics simulations. The chemokine receptor–chemokine interaction is highly stable and central, as indicated by both PPI network centrality and docking/MD analysis, and this could be a reason why chemokine receptor pairs are predominant in the maintenance of inflammatory signaling during the heat stress condition. The computational findings provide structural plausibility that complements the transcriptomic and network-level suppression of chemokine signaling following resveratrol treatment, thereby supporting the observation from the in vivo experiment. Even though docking and molecular dynamics studies indicate stable interaction of resveratrol with chemokine receptors, the current data cannot determine the mechanism as a competitive or allosteric modulation or some other special receptor regulation mode. Rather, these findings support the possibility that resveratrol may modulate chemokine receptor-mediated signaling, potentially in combination with broader antioxidant and transcriptional effects. Receptor-level modulation has been found to mitigate inflammatory responses across a variety of disease models with experimental inhibition of CXCR3 and CXCR4 signaling, thus showing a biological relevance of these receptors [62,63].

The combined results of our study support a model in which HS induces oxidative stress in CL, therefore promoting the activation of chemokine-centered inflammatory networks and ultimately impairing luteal structure and molecular integrity. Oxidative stress and inflammatory processes are increasingly recognized as closely connected in reproductive pathology, with ROS identified as an upstream modulator of inflammatory signaling [64,65]. Resveratrol may mitigate this pathogenic cascade by improving redox balance and inhibiting inflammatory network activation, thus helping to preserve the structural and molecular integrity of luteal tissue.

There are several limitations to consider. Although the current study establishes multi-level protection of the luteal tissue by resveratrol, it does not directly functionalize the chemokine receptor signaling or infiltration of the immune cells. Also, there was no resveratrol treatment group in this study, thereby limiting the ability to differentiate the actual transpired effects of resveratrol and the protective effects of resveratrol in heat stress conditions. The vehicle used to administer resveratrol was 0.5% carboxymethylcellulose, and there are no anticipated confounding effects, but the lack of a completely matched vehicle control within all groups must be considered when interpreting the findings. The whole ovarian tissue was used in transcriptomic and RT-qPCR analysis; hence, the molecular results could be due to combined ovarian responses and not only corpus luteum-specific actions. However, histological analysis was particularly directed at the corpus luteum to justify the luteal-related structural changes. Moreover, there was no measurement of functional endpoints, like the levels of progesterone and luteal endocrine activity, and thus, conclusions can only be made regarding structural and molecular changes. Further research needs to be conducted on the downstream signaling pathway, such as activation of NF-KB and STAT, and its impact on progesterone production and pregnancy outcomes under HS.

## 5. Conclusions

Heat stress induces oxidative, structural, and inflammatory alterations in luteal tissue in pregnant mice, characterized by redox imbalance and activation of cytokine–chemokine inflammatory networks. Resveratrol attenuates heat stress-induced oxidative, structural, and inflammatory alterations and partially preserves luteal tissue integrity. Biochemical analysis, transcriptomics, network biology, and molecular modeling enable the identification of the cytokine–chemokine receptor signaling as one of the key regulatory axes in heat stress-related luteal damage. These findings highlight the potential of resveratrol as a multi-target phytochemical for supporting luteal tissue integrity under heat stress conditions.

## Figures and Tables

**Table 1:** Primer sequences used in this study.

Gene	Gene ID	Primer Direction	Primer Sequence (5′–3′)	Product Size (bp)
*Ccr3*	XM_006522584.4	Forward	CGTGTTCAACATCCACAG	102
		Reverse	GCTGGTTGGTGATCTTCTGG	
*Ccr4*	XM_011242933.3	Forward	CCATTCTGGGGCTACTACGC	80
		Reverse	CAGCTCCTTGTTGCCATCCTG	
*Ccr5*	NM_009917.5	Forward	GTTGTTTTGGAGAACGCCCC	187
		Reverse	CAACACTGCTCCGAAACTGC	
*Ccl11*	NM_011330.3	Forward	AGCTAGTCGGGAGAGCCTAC	122
		Reverse	AAGGAAGTGACCGTGAGCAG	
*Cxcl13*	NM_018866.3	Forward	CTCTCCAGGCCACGGTATTC	140
		Reverse	CAGTTTTGGGGCAGCCATTC	
*G* *apdh*	NM_008084	Forward	AACTTTGGCATTGTGGAAGG	132
		Reverse	GGATGCAGGGATGATGTTCT	
*Tslp*	NM_001045528.3	Forward	CTGCAAGTCCACCTCTTCCT	98
		Reverse	ACAGGTACATCCATGAAACGA	

**Table 2:** Semi-quantitative histological scoring.

Group	Vacuolization (0–3)	Disorganization (0–3)	Inflammation (0–3)	Cell Density (0–3)	Overall Score (0–12)
Control	0.00 ± 0.00 ^a^	0.00 ± 0.00 ^a^	0.00 ± 0.00 ^a^	0.00 ± 0.00 ^a^	0.00 ± 0.00 ^a^
HS	1.40 ± 0.24 ^b^	1.60 ± 0.24 ^b^	1.80 ± 0.20 ^b^	1.60 ± 0.24 ^b^	6.40 ± 0.24 ^b^
HS + Resveratrol	0.60 ± 0.24 ^c^	1.20 ± 0.20 ^c^	0.60 ± 0.24 ^c^	1.00 ± 0.32 ^c^	3.40 ± 0.51 ^c^

Data are presented as mean ± SEM (n = 5 per group). Histological alterations, including vacuolization, disorganization, inflammation, and reduced cell density, were significantly increased in the HS group compared to the control and were attenuated by resveratrol treatment. Different superscript letters (a–c) within the same column indicate statistically significant differences among groups (*p* < 0.05), whereas identical letters indicate no significant difference.

**Table 3:** Docking parameters and interaction characteristics of chemokine–receptor complexes.

Parameter	CCL11–CCR3	CXCL12–CXCR4	CXCL10–CXCR3
Docking score	−304.06	−287.28	−268.69
Interface area (Å^2^)	1394.2	1337.2	1088.8
ΔiG (kcal/mol)	−21.3	−21.8	−20.3
ΔiG *p*-value	0.134	0.275	0.236
Salt bridges (n)	0	1	0
Disulfide bonds (n)	0	0	0
Hydrogen bonds (n)	0	1	1

**Table 4:** Binding energies and key interacting residues of resveratrol with chemokine receptors.

Receptor	BE (kcal/mol)	Interaction Category	Interaction Type	Distance (Å)	Key Residues
CCR3	−7.2	Hydrogen bond	Conventional H-bond	2.83	Phe126, Ile129, Leu164, Ala168
		Hydrophobic	π–π stacked	3.88	Phe126
			π–alkyl	4.77	Ile129
			π–alkyl	5.33	Leu164
			π–alkyl	5.04	Ala168
			π–alkyl	5.31	Leu164
CXCR3	−7.0	Hydrogen bond	Conventional H-bond	2.74	Asp51
			Conventional H-bond	2.35	Asp111
			Conventional H-bond	2.45	Ala112
		Hydrophobic	π–π stacked	4.56	Phe130
			π–π T-shaped	5.09	Asp185
			π–alkyl	5.44	Leu189
			π–alkyl	5.30	Trp108
			π–alkyl	5.40	Leu55
CXCR4	−6.8	Hydrophobic	π–sigma	3.82	Leu215, Leu223
			π–π stacked	4.27	Phe256
			π–alkyl	5.22	Ile252
			π–alkyl	5.04	Phe256

Notes: BE, binding energy (kcal/mol).

## Data Availability

The RNA sequencing datasets generated in this study have been deposited in the NCBI Sequence Read Archive (SRA) under BioProject accession number PRJNA1428318. The dataset includes 18 paired-end RNA-seq libraries derived from ovarian tissues of pregnant *Mus musculus* (ICR strain) assigned to control, heat stress, and resveratrol plus heat stress treatment groups. The data are publicly available at https://www.ncbi.nlm.nih.gov/bioproject/PRJNA1428318 (accessed on 26 February 2026).

## Abbreviations

ANOVA	Analysis of variance
BE	Binding energy
CL	Corpus luteum
DEGs	Differentially expressed genes
DPX	Distyrene plasticizer xylene
FDR	False discovery rate
GO	Gene Ontology
H&E	Hematoxylin and eosin
HS	Heat stress
KEGG	Kyoto Encyclopedia of Genes and Genomes
MCC	Maximal clique centrality
MD	Molecular dynamics
MDA	Malondialdehyde
NF-κB	Nuclear factor kappa B
NPT	Constant number of particles, pressure, and temperature ensemble
PCA	Principal component analysis
PPI	Protein–protein interaction
R+HS	Resveratrol + heat stress
RT-qPCR	Real-time quantitative polymerase chain reaction
RIN	RNA integrity number
RMSD	Root mean square deviation
RMSF	Root mean square fluctuation
RNA-seq	RNA sequencing
ROS	Reactive oxygen species
SEM	Standard error of the mean
STRING	Search Tool for the Retrieval of Interacting Genes/Proteins
T-SOD	Total superoxide dismutase
